# Supplementary material for: Remodeling mechanisms determine size distributions in developing retinal vasculature
Source: PLoS One. 2020 Oct 14;15(10):e0235373. doi: 10.1371/journal.pone.0235373 (PMC7556457; doi:10.1371/journal.pone.0235373)
Supplement: S1 File — (PDF) [file pone.0235373.s001.pdf]

Supporting Text

A. The implementation of Murray's law by the vessel radius growth function

By setting an appropriate function for vessel radius growth, a vascular network model obeying Murray's law can be generated. In the network model, vascular segment radii changed depending on their flow rate f and their own radii r as follows [6]:

$$\dot{r} = F(f) - dr. \tag{7}$$

F represents the growth function for vessel radii as influenced by the flow rate, and d represents the decline constant of the vessel diameters. Considering equilibrium states, $\dot{r}$ is zero, and we obtain

$$F(f) = dr. \tag{8}$$

By choosing the appropriate F, we can construct a vascular network that obeys Murray's law at a steady state. If a system obeys Murray's law, flow and diameter would have the following relationship:

$$f = ar^3. \tag{9}$$

By defining F as

$$F(x) = a^{-1/3}dx^{1/3}, \tag{10}$$

vessel segment radii at the steady state obey Murray's law (9).

We numerically confirmed this analysis (S1 Fig.). First, we prepared a tree-like network with equal radii. We set the inlet and outlet as the branch trunk and tip, respectively. We calculated the flows in each segment and changed the diameters according to $F(x)$ (S1 Fig. a). We confirmed that the bifurcated point obeys Murray's law (S1 Fig. b). Additionally, we showed that the segment number and radius show linearity on a log–log plot, with an inclination of -3 (S1 Fig. c).

B. Angle distribution and Murray's law

Murray provided another hypothesis for the optimal angles in the arterial trees [4]. He proposed that the bifurcation angles were also determined by an energy minimization argument. The expected angles were calculated using the following equation (S2 Fig. a):

$$\cos\theta_1 = \frac{r_0^4 + r_1^4 - r_2^4}{2r_0^2 r_1^2} \tag{11}$$

$$\cos\theta_2 = \frac{r_0^4 + r_2^4 - r_1^4}{2r_0^2 r_2^2}. \tag{12}$$

However, when we quantified the arterial tree angles, we identified a difference between the observed and predicted angles (S2 Fig. b). We defined Δ, the indicator of the difference, as follows:

$$\Delta = \sqrt{(\cos\phi_1 - \cos\theta_1)^2 + (\cos\phi_2 - \cos\theta_2)^2} \qquad (0 \leq \Delta \leq 2\sqrt{2}). \tag{13}$$

ϕ_1 and ϕ_2 were the observed angles and θ_1 and θ_2 were the predicted angles calculated from the radii r_0, r_1 and r_2. The highest peak of the distribution was approximately 1.4, and the value was large in this range. Contrary to our prediction, the peak did not decrease with the development of the artery. In the histogram of the real angles (S2 Fig. c), we observed the presence of two peaks at 10° and 80°. The first peak reflected the curve of thick branches, which had short branches (S1 Fig. d, arrows). The second peak reflected the random network generated by the astroglial Voronoi partition. Additionally, we observed that the peak did not decrease with the development of the artery.

C. Error estimation of mathematical morphology-based measurement

We used the mathematical morphology method (i.e., skeletonization and distance map) to estimate each blood vessel segment thickness. However, errors based on the lattice itself were present. For example, measuring a single vascular segment may be influenced by the angle between the segment and lattice. When the starting point of the segment

is origin $(0,0)$ and endpoint is (x,y), the real length L_{real} is expressed as

$$L_{\text{real}} = \sqrt{x^2 + y^2} \tag{14}$$

while the observed length, by counting skeletonized pixels, is expressed as

$$L_{\text{lattice}} = \max(x, y) \tag{15}$$

(Fig. S2). We examined the error of the blood vessel diameter measurement. First, we prepared an image set created by rotating an image of a straight line of a certain thickness. Because the real length and the thickness are identical in the rotated image, we were able to observe the influence on the segment diameter measurement due to the inclination. We prepared nine image sets of different angles θ ranging from $0 \sim 2\pi$ and measured the diameter employing the same method used to measure the diameter of the blood vessel (S3 Fig.). Although the number of lattices in the skeletonized segment depended on θ, we observed that the segment slope and error of the measured segment thickness were within 5%. In conclusion, the results of the present study demonstrate that in our method, the length error does not influence the radius measurement.

D. α-smooth muscle stain is suitable for estimation of the arterial radius.

We identified the retinal arteries with α-SMA stain (S4 Fig. ab). Because α-SMA stains vascular smooth muscle that surrounds the arterial wall, we may overestimate the arterial diameters. To rule out this possibility, we double-stained aSMA (smooth muscle) and IB4 (endothelium). The staining clarified that the arterial diameters observed using α-SMA stain were not very different from those stained using IB4 (Fig. S3b). To confirm this tendency, we plotted the relationship of diameters measured by α-SMA and IB4. Our results showed that the diameters measured using α-SMA and IB4 were almost identical (S4 Fig. c).

E. Variation of observed data

We used four retinas for each experiment. To show the variation of each sample, we plotted the average and standard error of the samples used in Fig. 2a, Fig. 3c, and Fig. 5b. Overall tendency is not different among samples. Fig 3. is the result of a stochastic mathematical model, and we can make standard error smaller by increasing the number of numerical simulations.

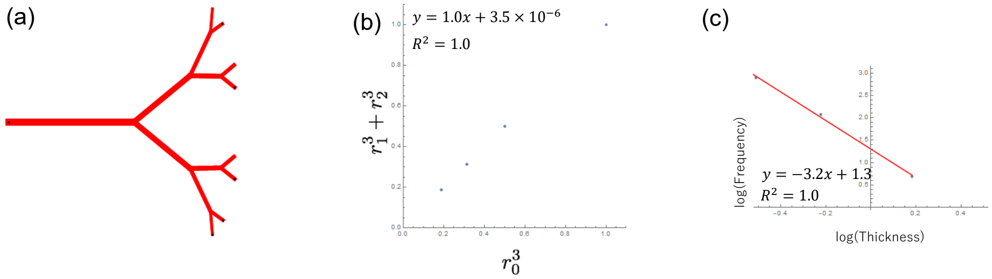

S1 Fig. Appropriate vessel diameter growth function can establish Murray's law. (a) Numerical simulation result of the branch with $F(x) = a^{-1/3}dx^{1/3}$. We predicted that if the growth function of the radius is $F(x) = a^{-1/3}dx^{1/3}$, Murray's law holds at the steady-state. (b) Relationship between $r_1^3 + r_2^3$ and r_3^3 in (a). Cubic sums appear equal, suggesting that these arteries obey Murray's law. (c) The log–log plot of blood vessel thickness and frequency in a numerical simulation. The gradient is close to -3, as predicted.

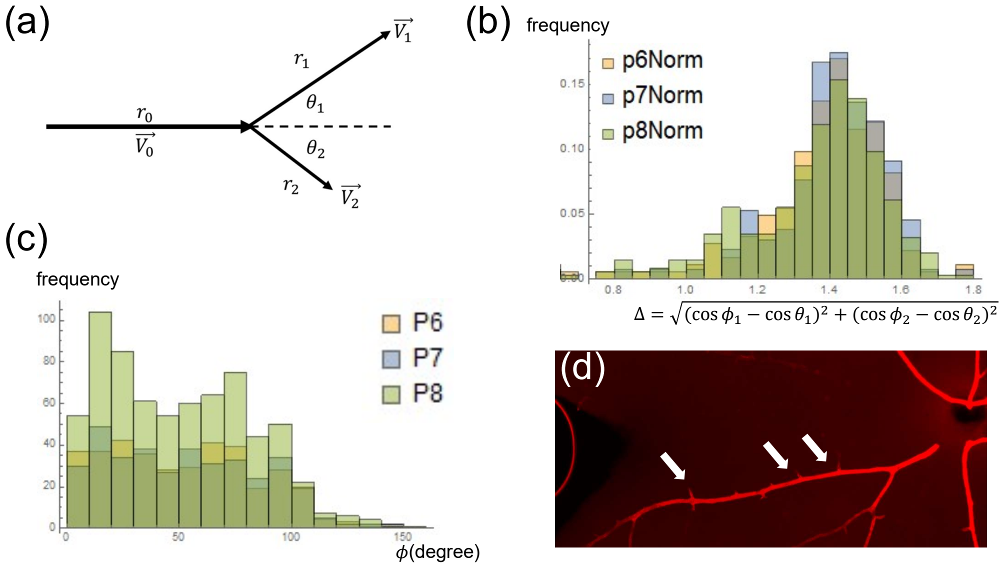

S2 Fig. Developing retinal artery angles do not obey Murray's law. (a) Definition of angles. $\vec{V_0}$ is the vector of the arterial segment before bifurcation. $\vec{V_1}$ and $\vec{V_2}$ are the vectors of arterial segments after bifurcation. r_0, r_1 and r_2 are the arterial segment radii. We assumed r_1 is larger than r_2. θ_1 represents the angle between $\vec{V_0}$ and $\vec{V_1}$. θ_2 is the angle between $\vec{V_0}$ and $\vec{V_2}$. (b) Difference between real and predicted values. (c) Distribution of real angles (ϕ_1 and ϕ_2). There are two peaks in the distribution of P8. The first peak is approximately 10°. The second peak is approximately 80°. (d) Image of arterial segments stained with α-SMA. The first peak reflects the bifurcation between the main large arterial trunk and a minor arteries (white arrows).

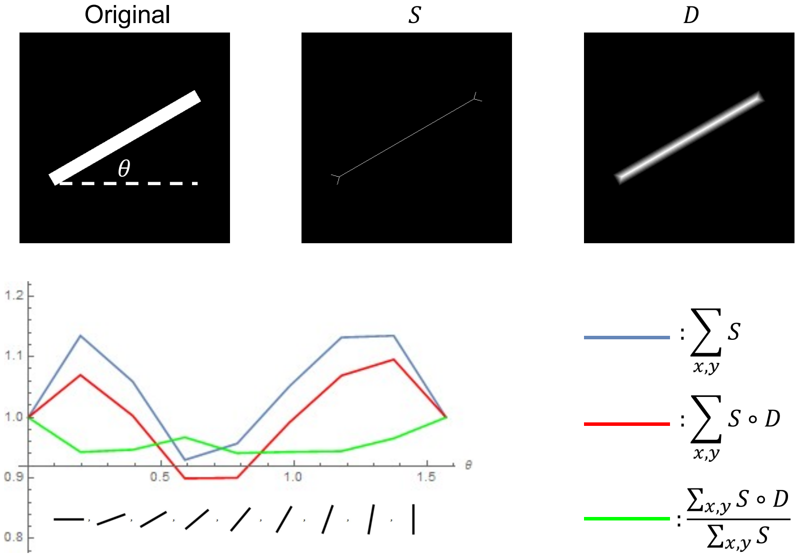

S3 Fig. Error estimation using lattice shape and segment angle. We prepared a skeletonized image S and distance map image D and calculated the segment length $\sum_{x,y} S$ and sum of segment thickness at the skeletonized region $\sum_{x,y} S \circ D$. After that, we obtained the thickness of the segment by dividing the sum of the segment thickness by segment length. Although the number of lattices in the skeletonized segment depends on θ (i.e., the segment slope), the error of measured segment thickness was within 5%.

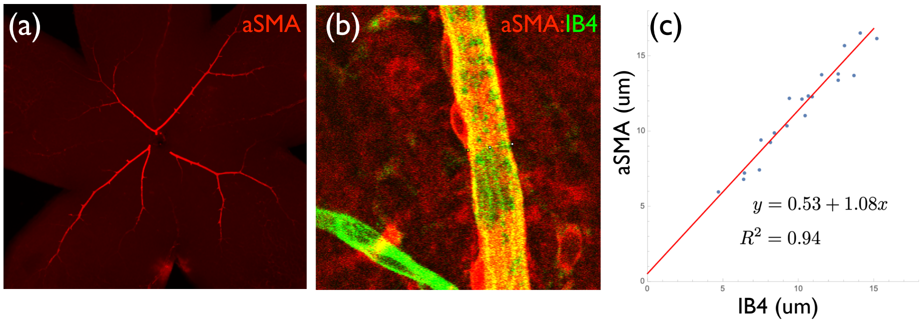

S4 Fig. Relationship between arterial diameters measured by smooth muscle and endothelial cell. (a) P8 retina stained by α-SMA. The arterial tree was stained in red. (b) Relationship between endothelial cells (green) and α-SMA (red) At this stage thickness of smooth muscle cell was very thin. (c) Relationship between diameter measured with α-SMA and endothelial cells. aSMA diameter is proportional to the endothelial cell diameter.

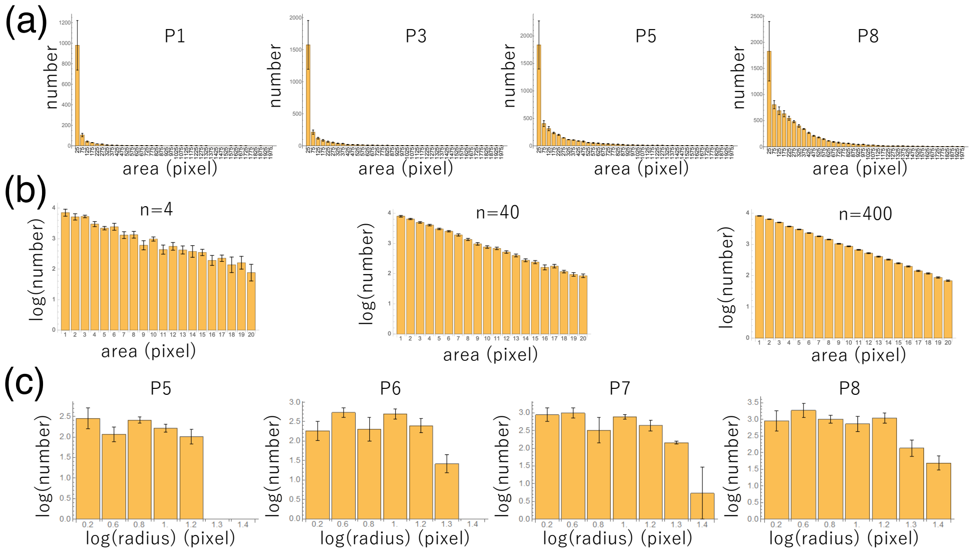

S5 Fig. Variation of measured vessel data among different samples. (a) Histogram of the island size at various developmental stages. Means and standard errors of 4 samples per each stage are shown. (b) Histogram of the island size distribution obtained by numerical simulations (sample size: $n = 4, 40, 400$). (c) Histogram of the average diameter of vascular segments in each sample. Means and standard errors of 4 samples per each stage are shown.
